# News Items

**Published:** 1917-05

**Authors:** 


### News Items.

### Over $17,000 Raised in Texas for Public Health Work.

Seventeen thousand one hundred and sixty-eight dollars and sixty-seven cents were the total gross receipts from the sale of Red Cross Christmas seals for the State of Texas as reported to the board of directors of the Texas Public Health Association at their annual meeting in Austin. Of this amount 10 per cent has been sent to the American Red Cross in Washington. The remainder, after paying the expenses of the sale, is to be used in anti-tuberculosis work in various cities and towns in Texas.

This association has recently been reorganized and has determined to carry on a very active campaign of education along public health lines. It was decided by the board of directors to employ a public health nurse to work in the smaller towns and villages of the State. She will be fitted to make an examination of the children in the public schools, when this is desired, and to investigate, make reports, and advise concerning improvements in public health and sanitary conditions in the towns which she visits. Mr. D. E. Breed, executive secretary of the association, whose offices are 616 Littlefield Building, Austin, Texas, will be glad to hear from any towns that are interested in securing her services.

It was also decided to have the association publish once a month a bulletin, for the benefit of the public, dealing with public health questions. This bulletin is to be particularly practicable for public school teachers and their pupils and may be had free of cost on application to Mr. Breed.

Mr. W. A. Bowen, Arlington, editor of the *Farmers' Fireside Bulletin,* was elected president for the ensuing year; Dr. Z. T. Scott, Austin, secretary, and Mr. H. A. Wroe, Austin, treasurer. The executive committee is composed of Mr. Bowen, Dr. Scott, Mr. J. D. Harper, Dallas; Mr. J. F. Marron, Austin, and Dr. B. T. Young, San Antonio.

It is the desire of this association to be of service to any local community. Application for co-operation along public health lines should be made to the executive secretary through the offices of the association.

### Planning Big Convention.

Plans for a largely attended convention of the State Medical Association, which will meet in Dallas, May 8-10, and for a program of unusual interest, are being arranged by the various committees. Dr. Samuel E. Milliken, chairman of the arrangements committee, reported that the details of the convention plans are being rapidly worked out. The sessions will be held in the Scottish Rite Cathedral.

---

### Infantile Paralysis Is Grip.

That even infantile paralysis is a form of the grip is the contention of Dr. Bernard Frankel, a New York physician, who, during the epidemic in New York last summer, did much research work in an effort to get at the cause of the disease. He contributed an article on the subject to the *New York Times,* in which he said:

"During last summer the grip did terrible execution among our children under the guise of the so much dreaded infantile paralysis, which I have found to be not a separate disease—as generally considered—but only a special form of grip, one that involved mainly the very vulnerable nervous system of children, thus giving rise to paralysis. All of its other symptoms were identical with those of other forms of grip, from which there was no possible way of differentiating it in the early stages before the very onset of paralysis. For this reason some physicians diagnosed during the height of the epidemic every severe case of grip as infantile paralysis. If, however, no symptoms of paralysis manifested themselves they called the case abortive, one that recovered before paralysis had had time to develop."

In support of his theory, Dr. Frankel refers to the findings of Dr. D. M. Lewis, bacteriologist of the New Haven Board of Health, who was enabled to trace the origin of last summer's epidemic of infantile paralysis in New Haven to the numerous preceding cases of grip among members of the same families in that community, and who also held that the so-called abortive cases of infantile paralysis were nothing else but typical cases of grip.

In reference to the terror spread among parents and children by the discovery that isolation was not a guarantee against contracting the disease, Dr. Frankel says:

"The rapid spread of infantile paralysis—even among families from which in their isolation all strange children were excluded for months—was attributed in a great measure to so-called 'carriers,' that is, persons who, while not suffering from the disease themselves, are supposed, nevertheless, to carry its germs about them, and thus communicate the disease to children with whom they come in contact. In my opinion these mysterious 'carriers' are ordinary cases of grip which communicate the infection to children in their immediate entourage. As a result of such infection infantile paralysis would develop in children predisposed to that disease, more especially under atmospheric conditions favoring such development. The practical application of this theory would imply the imperative necessity of extraordinary care in guarding children against any possible grip contagion, especially during the probable recrudescence of infantile paralysis during the coming summer; for, aside from the fact that grip is generally dangerous for children, they would also be threatened by possible paralysis complications in such seasons."

A scientific discussion of the relation of grip and infantile paralysis by the same author appeared in the *New York Medical Journal* December 2, 1916. In this article Dr. Frankel stated that he held that grip is caused not by Pfeiffer bacilli, but the same variety of streptococci that Dr. Rosenow recently discovered to be the cause of infantile paralysis, and that this view was supported by recent important observations of Dr. G. Moody and Dr. Matters of Chicago.

---

### Dr. C. M. Rosser Offers To Organize Base Hospital in Dallas.

Advices from Washington tell of the offer which has been made by Dr. C. M. Rosser of Dallas to organize and direct a base hospital in Dallas should such an offer meet the approval of the war department. Dr. Rosser wired Senators Culberson and Sheppard and Representative Sumners of this offer. The matter was laid before the proper officials and Dr. Rosser received a telegram from Surgeon General Gorgas of the United States Army saying the offer would be taken under consideration.

---

Beaumont physicians have shown their patriotism by volunteering to make free of charge all physical examinations and certificates for men who desire to enter the work in the navy yards and arsenals.

### Denison Physicians Called to Colors.

Dr. Chas. T. McGregor and Dr. A. M. Kahn have received messages directing them to report to the naval coast defense station at New Orleans for further orders.

They will be used in the base hospitals of the navy department. They will enter the service as assistant surgeons, with the rank of first lieutenant.

### Sealy Hospital Would Aid.

Realizing the imperative need for adequate hospital facilities which the government may be called upon to provide in the very near future, Dr. Edward Randall, president of the John Sealy Hospital Board, telegraphed Secretary of War Newton D. Baker, tendering the use of fifty beds and an efficient medical staff to care for that number of patients.

### Medical Students To Be Graduated.

The Medical Branch of the University of Texas has been requested to furnish the United States medical department of the army and navy as many doctors for service as is possible, and in response to this request, Dean W. S. Carter has announced that the present sophomore and junior classes of the University will not be given any summer vacation, other than one week, but will be called immediately to take up their junior and senior work with a view of graduating the junior class next January. The freshman class will not be affected by this decision upon the part of the faculty of the medical college.

The decision as announced by Dean Carter is subject to the approval of the Board of Regents of the University, but it is anticipated that there will be no objection to its being carried out in view of the existing need for more doctors in the military service.

Eleven members of the senior class are busy now reviewing their studies in preparation for the examination for entrance into the naval officers' reserve corps, which will be held some time next week. Dr. Carter has received advices from the medical board in Washington that a representative will reach Galveston about April 18 to hold the examination.

Following their acceptance, those entering the navy will be given certificates of graduation, Dr. Carter says, and will receive their diplomas at the regular graduation exercises on May 31.

---

### May Buy Hospital Train.

A new departure will probably be made by the War Department in the purchasing of the $90,000 hospital train which has been in service at Fort Sam Houston for the last ten months.

The train was rented by the United States government from the Pullman Company at the time of the mobilization of the National Guard last summer, on border points. It consists of ten Pullman cars, specially constructed for use as hospital cars. There is a cooking car, bed cars and nurses' apartment cars. The cars for the patients are equipped with side doors in order that stretchers may be carried through and the cars are furnished with single, iron beds for the patients.

A contract was made by the government with the Pullman Company whereby the monthly rental money would be applied on the purchase price of the train if the government should desire to buy it outright. At that time it was thought that the train might be needed only for a few months, but since the present crisis arose it has been thought advisable to buy the train.

Colonel Walter D. McCaw, chief surgeon of the Southern Department, urged that the train be purchased, as half of the purchase price already has been paid in rental to the Pullman Company. This will be deducted from the purchase price in case the government buys, which Colonel McCaw is sure will be done.

A conference of the chief surgeon, the chief quartermaster and the commander of the Southern Department resulted in a request being forwarded to the War Department for the purchase of the train.

---

### Baylor Medical Staff Ready To Aid In War.

An offer to the government of the services as an organization of the clinical staff of Baylor University college of medicine, constituting the staff of the Texas Baptist Memorial Sanitarium, for the early development of a base hospital which can be used for convalescents or active service, in case fighting should be sufficiently near Dallas for such use, has been made. Dr. Edward H. Cary, dean of the college, announced that the staff had decided to follow the example of Johns Hopkins, Western Reserve University, and other leading medical schools, co-operating through the boards of directors of their hospitals. He also announced the medical school will remain open all summer.

Dr. Cary said: "This action also contemplates the development of a unit which the government can move to any other point desired, in the care of its forces. It is expected that the board of directors of the Baptist Sanitarium will actively support any effort on the part of its staff to help the government.

"The medical school has been requested to remain open throughout the summer and continue its work. The students have been urged by the dean of the medical department not to join any sanitary or other department of the government for immediate service because a pressing need is now felt for medical men, as it is estimated that 10 per cent of the doctors in the United States will be needed if the contemplated army is put in service. Hence the desire of the government to have the men who are now studying medicine and others who might be sufficiently well educated to commence the study of medicine to pursue this object of becoming doctors so that the medical ranks can be recruited with modernly educated men.

"This whole thing contemplates a change that will eliminate the present four months of summer vacation and will make it possible for the new term to commence the first of June. The City Hospital of Dallas as well as the Baptist Sanitarium offers fine clinical training."

### New School For Nurses.

The contract for the building of the nurses' training school, to be erected east of the main building of the Baptist Sanitarium, has been let, and actual construction work will begin immediately.

The contract calls for the completion of the structure by December 1, at a cost of $125,000. With furnishings, the school will cost approximately $160,000.

### Sanitarians Needed In U. S. Army Service.

Dr. W. S. Leathers, Director of Public Health of Mississippi, has advised Dr. W. B. Collins, Texas State Health Officer, that sanitarians and sanitary engineers are needed badly by the American army. He asks that the Southern States be circularized in the interest of procuring persons qualified to conduct this important work. Dr. Leathers may be addressed at the University of Mississippi at Oxford.

---

The Baptists of San Antonio are planning a $65,000 sanitarium, and more than half of the funds have already been subscribed.

---

### Dr. Carrel of France To Teach American Physicians And Surgeons the Wonders Of War Surgery.

All the marvels of the military surgical skill of Dr. Alexis Carrel and Dr. H. D. Dakin, who are recognized as the leading practitioners of military surgery in the world today, are about to be placed at the disposal, so far as such technical knowledge and expertness can be imparted to others, of the army and navy surgeons of the United States. Two hundred thousand dollars has be appropriated by the Rockefeller foundation to bring Dr. Carrel and Dr. Dakin to New York to equip a hospital for them and to open their clinics to classes of American army and navy surgeons.

The Rockefeller foundation further announced that preliminary arrangements and the complete organization of the new instructive unit have been completed, so that Dr. Carrel and Dr. Dakin, as soon as they have completed the necessary formalities of obtaining leaves of absence from the French government, will begin the instruction of classes of American surgeons who will care for our wounded in case of war with Germany.

### HOSPITAL READY IN TEN WEEKS.

The hospital, it is expected, will be ready within ten or twelve weeks. Charles Butler, who has made a thorough study of the war time hospitals of France and England, will have charge of the portable unit, which will have accommodations for one hundred wounded men. All the regular work of a hospital will be undertaken at the new unit, but its chief object will be to make it first and last an institution of experiment and instruction.

The hearty co-operation of the army, the navy and of the public health officials with the Rockefeller institute and the two surgeons has already been assured. The hospital will consist of several detached one-story wooden buildings, which can be completed quickly. As soon as these buildings have been erected Dr. Carrel and Dr. Dakin will open their laboratories on the grounds and begin work.

The great success of Dr. Carrel and Dr. Dakin in their Compiegne Hospital since the outbreak of the great war, especially in the advances made by Dr. Carrel in the treatment of deeply wounded legs and arms which formerly were invariably amputated, but now are saved by the new Carrel treatment, is widely known even among laymen.

In bringing Dr. Carrel to this country the Rockefeller foundation has, in addition to the general idea of patriotic service, three specific objects in view. As listed by the foundation, these objects are:

The make available to patients the improved method of treatment.

To demonstrate and teach the American surgeons who may be enrolled for military service measures for the treatment of infected wounds.

To test the feasibility of a portable military hospital unit.

It is planned to have the American surgeons who are to study the new methods of military surgery assigned to the hospital in successive groups. Each group of student surgeons will spend a minimum period of four weeks studying the method of application and the results obtained.

### Personals.

Dr. Youngblood of Falls City, accompanied by Mrs. Youngblood, is in Chicago doing post-graduate work in the Chicago Polyclinic and Hospital.

Dr. Goodall Wooten has just returned from New York, where he went to place his daughter, Lucy, in school. While in New York, Dr. Wooten witnessed some very interesting operations.

Dr. R. C. Black has moved from Bluffdale to Detroit.

The Santa Fe Railway Hospital Association has elected Dr. A. C. Scott, chief surgeon; Dr. M. W. Sherwood, assistant chief surgeon; Dr. O. F. Gober, chief physician,—all of Temple and at present connected with the railway hospital there.

In last month's issue of the JOURNAL we had an article on "The Pre-Cancerous Cervix," which we said was written by S. A. Cunningham, M. D. This was an error. It should have been S. P. Cunningham, M. D., of San Antonio.

Dr. Collins of Austin, State Health Officer and president of the

State Board of Health, has issued a call for a meeting of the State Board and officials of the public health societies of Texas to meet in Dallas on May 7 for the purpose of discussing with a representative of the American Public Health Association a plan of consolidating all societies.

The annual convention of the State Nurses' Association was held in Temple, April 17.

The fifty-first annual meeting of the State Medical Association of Texas will be held in Dallas, May 8, 9 and 10. Many prominent physicians and surgeons of the United States are on the program.

The Jones County Medical Society met in regular session in Stamford, April 10. A very interesting program was rendered, including a paper by Dr. Leggett of Abilene.

Dr. J. H. Thomas, formerly of Doucette, has located at Orange.

Dr. W. H. McRae of Fort Worth recently sustained painful injuries from a fall received while getting on a street car. The car started off too soon, throwing him to the ground.

For the second time since the organization of the Central London Throat and Ear Hospital in 1874 an American has been appointed house surgeon. This honor was conferred on Dr. Roy Dunlap, whose parents live in Fort Worth. Dr. Dunlap sailed from New York November 4, 1916, for London, attended clinics and took a course in the Central London Ear and Throat Hospital. It is the purpose of Dr. Dunlap to remain in London for an indefinite period and practice and study in the hospital there to perfect himself in his profession.

---

### Deaths.

Dr. G. W. Baskett, a prominent physician of Van Alstyne, died at his home in that city recently.

Dr. C. B. Clark of Ardmore, Oklahoma, died recently at Allison Bros.' Sanitarium, Fort Worth, of pneumonia.

---

Typhoid fever is a disease peculiar to man.

---

"Did you get acclimated when you went to Cuba?"

"Yes, and by the best doctor I could find, but it didn't take."

